# Exploring the Potential of Broadband Complementary Metal Oxide Semiconductor Micro-Coil Nuclear Magnetic Resonance for Environmental Research

**DOI:** 10.3390/molecules28135080

**Published:** 2023-06-29

**Authors:** Daniel H. Lysak, Marco Grisi, Kathryn Marable, Gaurasundar M. Conley, Carl A. Michal, Vincent Moxley-Paquette, William W. Wolff, Katelyn Downey, Flavio V. C. Kock, Peter M. Costa, Kiera Ronda, Tiago B. Moraes, Katrina Steiner, Luiz A. Colnago, Andre J. Simpson

**Affiliations:** 1 Environmental NMR Centre, University of Toronto, Toronto, ON M1C 1A4, Canada; 2 Annaida Technologies, Innovation Park, 1015 Lausanne, Switzerland; 3 Department of Physics and Astronomy, University of British Columbia, Vancouver, BC V6T 1Z1, Canada; 4 Departamento Engenharia de Biossistemas, Universidade de São Paulo/ESALQ, Av. Páduas Dias, 11, Piracicaba 13418-900, SP, Brazil; 5 Embrapa Instrumentação, Rua XV de Novembro 1452, São Carlos 13560-970, SP, Brazil

**Keywords:** micro-coil NMR, environmental NMR, CMOS, heteronuclei, steady state free precession

## Abstract

With sensitivity being the Achilles’ heel of nuclear magnetic resonance (NMR), the superior mass sensitivity offered by micro-coils can be an excellent choice for tiny, mass limited samples such as eggs and small organisms. Recently, complementary metal oxide semiconductor (CMOS)-based micro-coil transceivers have been reported and demonstrate excellent mass sensitivity. However, the ability of broadband CMOS micro-coils to study heteronuclei has yet to be investigated, and here their potential is explored within the lens of environmental research. Eleven nuclei including ^7^Li, ^19^F, ^31^P and, ^205^Tl were studied and detection limits in the low to mid picomole range were found for an extended experiment. Further, two environmentally relevant samples (a sprouting broccoli seed and a *D. magna* egg) were successfully studied using the CMOS micro-coil system. ^13^C NMR was used to help resolve broad signals in the ^1^H spectrum of the ^13^C enriched broccoli seed, and steady state free precession was used to improve the signal-to-noise ratio by a factor of six. ^19^F NMR was used to track fluorinated contaminants in a single *D. magna* egg, showing potential for studying egg–pollutant interactions. Overall, CMOS micro-coil NMR demonstrates significant promise in environmental research, especially when the future potential to scale to multiple coil arrays (greatly improving throughput) is considered.

## 1. Introduction

NMR is a powerful analytical technique used for everything from confirming an organic synthesis product [1], to analyzing complex mixtures [2] or even studying living organisms [3]. NMR gives an unparalleled molecular level understanding of a sample in a non-invasive fashion. However, the key challenge of NMR is sensitivity. There has been much work in improving NMR sensitivity in general (e.g., cryoprobes [4,5], stronger magnets [6], hyperpolarization techniques [7]), but for tiny, mass limited samples, the use of micro-coils can be an excellent choice [8]. Micro-coils with sizes in the range of 100 s of microns have been reported to have mass sensitivity values orders of magnitude higher [9,10,11] than standard size (e.g., 5 mm probes), allowing for greatly improved NMR analysis of tiny samples. 

This benefit has been leveraged in the past to study samples such as micron-sized eggs [12,13,14], which can be crucially important to environmental health. As an example, *Daphnia*, the most commonly studied organisms in aquatic toxicity testing [15,16,17], and an NIH model organism for the study of human diseases [18], has the potential to lay resting eggs when environmental conditions are unfavorable [19]. These eggs can persist in sediment for several years [20] in order to “wait out” unfavorable conditions (e.g., low water temperature), and hatch when conditions improve. However, if during this time the eggs are wiped out by exposure to anthropogenic pollutants, the consequences can be disastrous. Indeed, *D. magna* is an ecological keystone species—a crucial link between producers such as algae and consumers such as small fish, and is critical to the ecological health of the environment [21,22,23]. Understanding the impacts of pollutants on *D. magna* in various life stages (eggs, neonates, adults) is therefore of significant value. 

Traditional (acute) toxicity testing usually involves an examination of the apical endpoints, such as when an organism stops moving, is unable to reproduce or dies [24]. However, this is challenging when studying eggs as they do not move or reproduce for extended periods of time [14]. Further, this does not provide information on sublethal toxicity which is more likely to occur in the environment [25]. As such, the molecular level understanding that can be provided via NMR is a valuable asset. However, studying individual eggs (which range in size between ~200–500 μm) on standard-sized probes is very difficult and filling a standard 5 mm coil could require thousands of eggs for a single experiment [13]. On the other hand, the improved mass sensitivity makes a micro-coil perfectly suited to the analysis of individual eggs. 

A recent trend in micro-coil NMR has been the use of complementary metal oxide semiconductor (CMOS)-based microchips which include an integrated circuit containing all transceiver electronics [26,27,28,29]. Compared to standard micro-coils which have discrete electronic components, CMOS chips have been reported to have several advantages including excellent sensitivity due to the proximity of the coil to the preamplification elements. This results in very little signal loss due to transmission lines and decreased parasitic losses due to the elimination of coil leads. Further, CMOS chips allow for easy expansion to multiple coils on a single chip for improved throughput and the ability to have a broadband coil for analysis of heteronuclei [30,31,32,33]. CMOS-based NMR sensors have previously been used in the literature precisely for the ^1^H analysis of eggs in the nL and sub nL volume range [12,34]. However, the broadband potential of these chips has yet to be explored. 

Here, the potential of a broadband, CMOS-based micro-coil sensor is explored within the context of environmental research. First, a series of chemical standards are analyzed to establish lineshape and sensitivity of various heteronuclei. Following this, two environmentally relevant samples are studied: a sprouting ^13^C labelled broccoli seed and a single *Daphnia magna* egg. A ^13^C steady state free precession (SSFP) sequence was used to improve the signal-to-noise ratio in the broccoli seed. ^1^H NMR was used to examine the initial metabolite profile of the *D. magna* egg, and ^19^F NMR was used to track a fluorinated contaminant within the single egg. Overall, the goal of this work is to answer the question: “does CMOS-based micro-coil NMR hold potential for environmental research?”

## 2. Results and Discussion

### 2.1. Exploring Heteronuclei

One of the major advantages of the CMOS micro-coil system employed here is that it is broadband over a wide frequency range. Thus, in contrast to commercial micro-coils which are typically tuned to a single nucleus, most commonly ^1^H, the CMOS technology used here is capable of analyzing a variety of nuclei and provides additional molecular insight versus using ^1^H NMR alone. Figure S1 shows spectra of eight heteronuclei that were successfully detected using the CMOS micro-coil system. These nuclei were chosen as they each have significant potential for the study of either environmental pollution or biochemical changes. As an example, ^7^Li is a major constituent of lithium-ion batteries [35]. Given the global shift towards electric vehicles, global lithium production is drastically increasing, and begets the issue of lithium pollution [36,37], which can be studied using ^7^Li NMR. ^31^P is a key component of biochemical energy molecules such as adenosine triphosphate [38,39,40] and ^31^P NMR can also be used to study biogeochemical phosphorus cycling [41,42]. ^205^Tl is a biological mimic for potassium [43] used in protein channel studies [44,45] and is a particularly interesting nucleus from an instrumentation perspective. The resonant frequency of ^205^Tl is in a “no man’s land” resonating above ^31^P (typically the uppermost frequency of broadband probes) but below ^19^F which is the next commonly studied nucleus. As such, this frequency is outside the range of nearly all commercial NMR probes, while the micro-coil system can study this nucleus without problems. 

### 2.2. Limits of Detection for Heteronuclei

Table 1 shows the approximate limits of detection for a 48-h experiment for the nuclei studied. Though this table is meant to be a general reference for the CMOS micro-coil detection limits, it is important to note that these values are calculated from simple standard solutions, which can have significantly better lineshape than more challenging samples. This is especially important for quadrupolar nuclei where the symmetry of the compound being studied can significantly impact the lineshape [46], and limits of detection for asymmetric compounds can be substantially higher [47]. Despite these limitations, it can be seen that the heteronuclei studied here have detection limits in the mid to high picomole range. Due to the importance of carbon for environmental analysis, instead of testing on a standard, a natural sample is considered in the next section.

### 2.3. ^13^C Analysis of a Broccoli Seed

**Figure 1:**
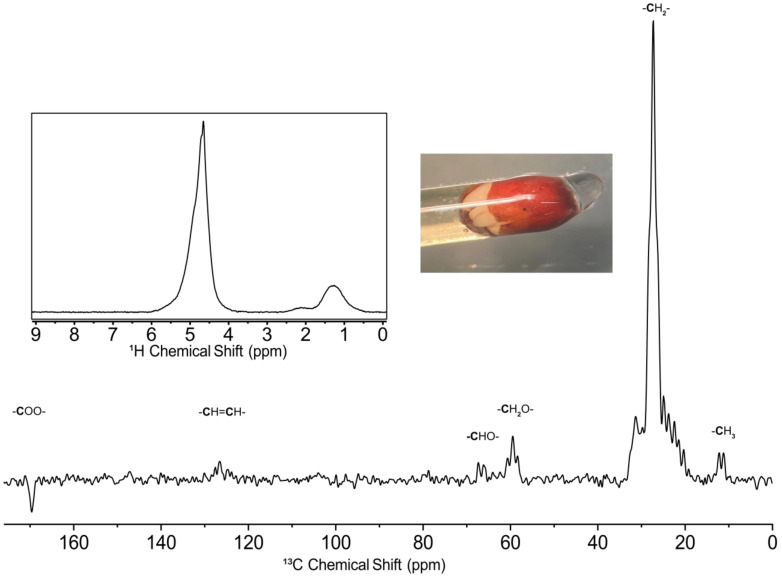
Steady state free precession ^13^C spectrum of a ^13^C labelled broccoli seed acquired at 11.7 T (500 MHz ^1^H). The inset shows a ^1^H NMR spectrum of the same sample, and a photograph of the sprouting broccoli seed in the thin-walled capillary used for analysis. The bold denotes the atom that gives rise to the resonance.

Since all organic compounds are carbon-based by definition, ^13^C NMR is a powerful tool that can be highly complementary to ^1^H NMR. The ~200 ppm wide chemical shift range of ^13^C allows for improved spectral dispersion which can be useful for components that may not be easily resolved in ^1^H NMR [48]. This is especially true for an intact sample such as the broccoli seed used here, as the presence of solid/semi-solid components creates magnetic susceptibility mismatches [49]. This results in broad lineshape and thus ^1^H NMR (see the inset in Figure 1), which only spans ~12 ppm, can be very challenging to interpret due to spectral overlap, and the enhanced dispersion of ^13^C is desirable. However, ^13^C NMR is significantly less sensitive than ^1^H [46], and often isotopic enrichment is required for the study of complex samples. Here, a ^13^C enriched broccoli seed was studied and the resultant steady-state free precession spectrum can be found in Figure 1. In simple terms, SSFP is a way to optimize the signal-to-noise ratio per unit time in NMR and is used widely in magnetic resonance imaging (MRI) [50]. Comparison to standard ^13^C NMR and more discussion is provided in the next section. Since this is a solution state experiment, there is a bias towards more mobile compounds, and the ^13^C signals arise mainly from mobile lipids in the broccoli seed. In order to supply the energy required for initial growth, seeds are known to store large quantities of lipids, which contribute to these signals [51]. It can also be noted that the peak at ~170 ppm is inverted. This is due to bandwidth limitations, as during setup the authors were not sure what bandwidth the SSFP sequence would be able to excite and therefore chose to set the transmitter frequency close to the largest peak (-**C**H_2_- region) to be safe. 

### 2.4. SSFP Compared to Standard ^13^C NMR

Due to the sensitivity challenges of ^13^C NMR, the SSFP pulse sequence was utilized to improve the signal-to-noise ratio. A standard pulse acquisition experiment uses a recycle delay which allows for excited nuclei to relax to thermal equilibrium in between successive scans. For 1D NMR this is commonly set to 3–5 times the T_1_ of the sample being studied, which can be 5–10 s or longer for ^13^C experiments. On the other hand, SSFP works by pulsing faster (often with a small flip angle pulse) than the relaxation time of the sample, which results in a steady state of magnetization that can be measured [52]. In practice, the time between successive pulses can be on the order of 10s of milliseconds which allows for a much larger number of scans to be acquired. This requires the acquisition time to be short (i.e., few points acquired) and thus there is a cost in spectral resolution. However, if the signals are already broad due to the nature of the sample (as is the case here with the broccoli seed), the resolution cost can be minimal, while still providing a significant gain in signal-to-noise ratio. 

**Figure 2:**
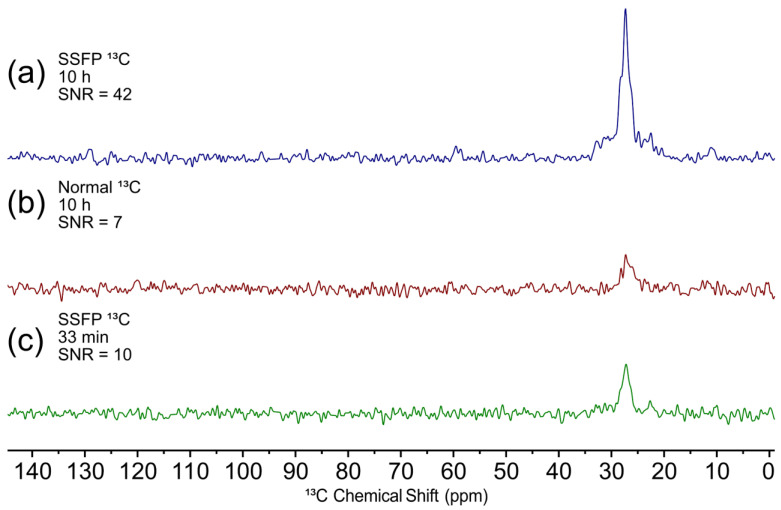
A comparison of: (**a**) a 10 h SSFP spectrum of a ^13^C enriched broccoli seed, (**b**) a 10 h standard ^13^C spectrum and (**c**) a 33-min SSFP spectrum. All spectra were acquired at 11.7 T (500 MHz ^1^H).

Figure 2a shows a 10 h SSFP ^13^C spectrum and Figure 2b shows a 10 h standard ^13^C SSFP spectrum of the broccoli seed. The SSFP spectrum has a six-fold higher signal-to-noise ratio and it is possible to see the formation of additional peaks near 10 and 60 ppm which are completely absent in the standard spectrum. To show the time savings that could be achieved using this approach, Figure 2c shows a 33-min SSFP experiment which still has a higher signal-to-noise ratio compared to the standard spectrum, despite being ~20 times faster to acquire. Further, it can be seen that the loss in resolution is at most minimal, making SSFP a powerful approach to improve the signal-to-noise ratio on “challenging” samples. In the future, this could be used to examine biochemical changes during germination in an individual seed, or study ^13^C labelled *D. magna* eggs. 

### 2.5. ^1^H Analysis of a D. magna Egg

**Figure 3:**
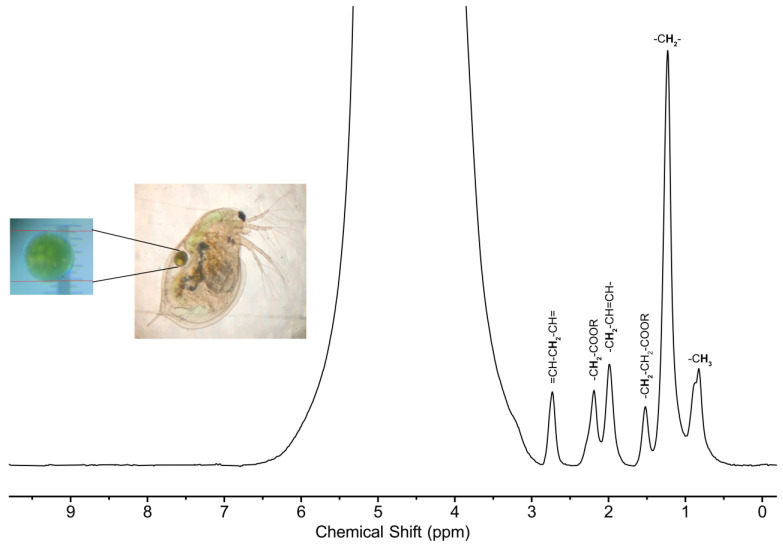
^1^H NMR spectrum of a single *D. magna* egg acquired at 500 MHz. The inset shows a photograph of an adult *D. magna* and its egg. The bold indicates the atom giving rise to the resonance.

A *D. magna* egg is an excellent example of a sample that is best studied using micro-coils as opposed to standard 5 mm probes. Since the volume of the egg is on the order of tens of nanoliters, filling a 5 mm probe is not feasible as it would require thousands of eggs. On the other hand, the CMOS micro-coil system can analyze a single egg as seen in Figure 3. However, an egg is a much more challenging sample compared to a capillary filled with a standard solution. First, it is more than 90% water by mass [53], and this leads to a very large water peak in the spectrum at ~4.7 ppm. This peak can mask the presence of less concentrated metabolites and it is difficult to resolve peaks between 3–6 ppm in the spectrum. Further, though to a lesser extent than the broccoli seed, the non-uniform composition of the egg introduces magnetic susceptibility differences within the sensing volume. Essentially, different parts of the egg experience slightly different magnetic fields, which, when summed over the entire sample volume, lead to significant lineshape broadening. While a ^1^H lineshape of ~4 Hz (~8 ppb) is achievable on a solution in a thin-walled capillary, this value jumps to ~50–100 Hz for an egg. 

Despite these challenges, it is still possible to resolve a number of peaks corresponding to lipid signals which are assigned in the spectrum. Fatty acids, which are key components of lipids, have been proposed as biomarkers for ecological food-web assessments and tracing [54]. As an example, *D. magna* are unable to synthesize long chain poly unsaturated fatty acids (PUFAs) de novo [55], and must acquire these components from their diet [56]. Using these compounds as biomarkers can shed light on the dietary behavior of the species. Further, changes in these lipid signals have previously been implicated in various metabolic changes and processes. In unfavorable conditions such as starvation, poor food quality or elevated temperature, *D. magna* stockpile PUFAs while saturated fatty acids are metabolized [57]. This could be identified in the ^1^H NMR spectrum by an increase in the =CH-C**H**_2_-CH= peak and a decrease in the -C**H**_2_- peak. Outside of the environmental field, changes in the ratios between PUFAs and saturated fatty acids have been associated with increased apoptosis in glioma cells [58]. As such, there is considerable future potential to examine the health of small eggs/organisms using CMOS micro-coils.

### 2.6. ^19^F Contaminant Tracking in a D. magna Egg

^19^F is a key component of many pollutants including the poly and perfluoroalkyl substances (PFAS). These compounds are used in a wide range of consumer products such as non-stick cookware, firefighting foams, surfactants and lubricants, mainly for their non-stick and water repelling properties [59]. However, due to the strength of the carbon fluorine bond, PFAS compounds are extremely persistent in the environment, earning the moniker of “forever chemicals” [60]. Recent studies have shown that PFAS are widespread in the environment and have been reported in surface waters [61], arctic snow [62], the top of Mount Everest [63] and even human blood and tissue [64]. PFAS compounds are known to bioaccumulate in fish and wildlife and thus the ability to study ^19^F in *D. magna* eggs can be of significant value [60]. Further, ~25% of pharmaceuticals and numerous agrochemicals contain ^19^F [65], and there is potential to study the binding and interactions of these compounds in *D. magna* eggs, which can shed light on toxic modes of action [66]. 

**Figure 4:**
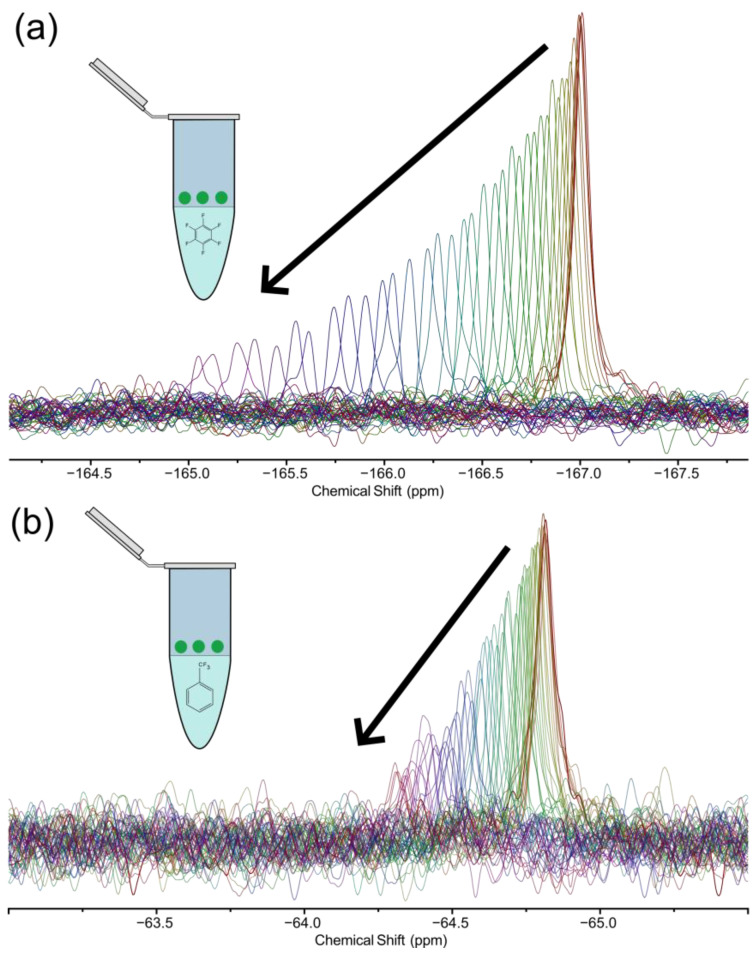
A time series tracking (**a**) hexafluorobenzene and (**b**) trifluorotoluene in a single *D. magna* egg. Each experiment was acquired at 500 MHz. The first spectrum is the thicker orange line and the black arrows indicate the direction of the subsequent spectra in the time series. To maintain clarity, every second spectrum in the time series is shown. The inset shows a schematic of the setup for overnight exposure.

As a proof of concept, Figure 4 shows a ^19^F time series tracking the fluorine signal in a *D. magna* egg exposed to hexafluorobenzene and trifluorotoluene. Each experiment in the time series was 6.3 min long, and thus the time resolution of the experiment is very good, especially considering the very low sensing volume of ~11 nL. This experiment time was chosen to ensure that a clear trend could be followed, and that the signal did not drop below the limits of detection for a few hours. However, an initial ^19^F signal could be detected after four scans (6 s) for hexafluorobenzene and 16 scans (24 s) for trifluorotoluene, demonstrating the excellent sensitivity of this nucleus even for a challenging sample, and showing that there is considerable potential for ^19^F tracking in single eggs in the future. 

Since the egg was thoroughly washed in order to remove any fluorinated compound that may adhere to the outside, and placed in fresh water, the fluorinated signal truly arises from the compound that has accumulated inside the egg during the overnight exposure. Throughout the time series, both compounds follow the same general pattern; the signal intensity is highest at the beginning of the time series and decreases as time progresses, and the decrease is associated with a change in chemical shift. This decrease in signal intensity could be attributed to either contaminant binding in the egg (for example to proteins, as the strong chemical shift anisotropy of the ^19^F nucleus would cause bound fluorine signals to broaden out to the point of being undetectable [67]), or partitioning of the contaminant out of the egg followed by diffusion outside of the sensing region. 

In order to ascertain which of these occurred, 10 eggs were prepared as described in the Materials and Methods section but placed in a small volume (~300 μL) of fresh water. After leaving these eggs overnight, they were removed and the water was analyzed on a cryoprobe, and it was found that the fluorinated compound indeed leached into the surrounding water. Though initially counterintuitive as both compounds are expected to be more soluble in a lipid medium (i.e., the egg cell membranes) compared to water, the drastic difference in volumes between the egg and surrounding water explains this behavior. The *D. magna* egg has a volume of ~20–30 nL but is placed in the egg holder which has a hydration chamber filled with ~1 mL of water. Considering the K_ow_ of trifluorotoluene is reported by the manufacturer as ~1000, even in the “worst case scenario” where the egg was assumed to be pure octanol, it is expected that ~97% of the initial mass of trifluorotoluene would partition into the surrounding water at equilibrium, explaining the drop in signal intensity. Even more would be expected to partition out for hexafluorobenzene, which is less hydrophobic and has a K_ow_ of ~350.

There are a number of differences between the time series for hexafluorobenzene and trifluorotoluene. First, the initial signal intensity is higher for hexafluorobenzene, which is due to the fact that it has six magnetically equivalent ^19^F atoms, while trifluorotoluene has only three, and thus each molecule of hexafluorobenzene contributes twice as much signal compared to trifluorotoluene. However, despite having a lower initial intensity, the ^19^F signal remains detectable for longer for trifluorotoluene, as can be seen by the additional spectra in Figure 4b. The half-life for signal decay is around 4.3 h for hexafluorobenzene but around 50% longer for trifluorotoluene (6.5 h). This can be expected as trifluorotoluene is more hydrophobic, and it has been reported that compounds with higher K_ow_ show slower outflow kinetics in aquatic invertebrates and fish [68]. Further, the -CF_3_ group, which is present in trifluorotoluene, has been previously reported to be involved in the non-covalent binding of pesticides and pharmaceuticals [69]. As such, there is considerable potential to study interactions of fluorinated compounds within single eggs using this approach. 

## 3. Materials and Methods

**Figure 5:**
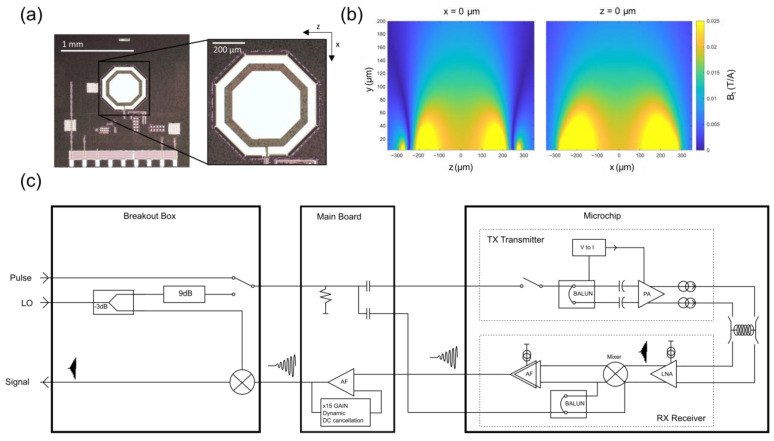
(**a**) Photograph of the CMOS chip and inset of the coil (30A is shown). (**b**) A map of the B_1_ field produced by a 1 A current through the coil as computed by the Biot Savart law, using a circular approximation for the octagonal micro-coil loops. (**c**) A block diagram of the major components of the micro-coil transceiver. The signal originates in the micro-coil and it is amplified and down-converted before transmission to the main board (a printed circuit board having approximate dimensions of 6 cm × 2 cm). The main board has the function to transmit all inputs to the sensor header and dynamically cancel any DC present in the sensor output before further amplification. The signal is then transmitted to a breakout box where it can be upconverted to its original frequency for full compatibility with standard NMR consoles (i.e., Bruker in this case).

All spectra were acquired on a 500 MHz Bruker Avance III spectrometer using a custom-made CMOS micro-coil device (Annaida Technologies, Switzerland). The microchips are produced using a standard 180 nm CMOS process by Taiwan Semiconductor Manufacturing Company (TSMC). Figure 5a shows a microchip used in this study (series 30A). The microchip area is 2 mm × 2 mm, containing a micro-coil composed of 11 loops realized using the 2 metal layers available in the CMOS technology. The coil is broadband with a self-resonance at about 650 MHz. The outer and inner diameters are, respectively, ID = 400 μm, OD = 490 μm. A second microchip (series 23B) was used to analyze the egg as this has a built-in egg holder (see later). In this case, the coil is made by stacking 5 metal layers and connecting them through vias to create 7 loops. The outer and internal diameters are, respectively, ID = 380 μm, OD = 560 μm. 

Inside the microchip, the micro-coil is switched between the integrated transmitter and receiver during the different steps of the measurement, similarly to previous work [28]. In the transmitter, an incoming pulse supplied by the external Bruker console is converted to a differential signal amplified with a power amplifier so that maximum current can be provided to the coil. In the receiver, the signal is first amplified by a low noise amplifier, then mixed down to audio frequency before a second amplification stage in the audio-frequency range. The total gain inside the microchip is 54 dB. The power consumption of the whole receiver is below 4 mW, while the transmitter consumes about 25 mW in transmit mode, and it is shut down to consume nearly zero power in receive-mode. These consumption properties ensure that the heat generated by the microchip cannot impact in-vivo samples during the measurement. The signal passes through a main board that has additional amplification as well as cancellation of the DC offset, then is mixed up to the original frequency using a frequency mixer (Minicircuits ZLW-1-1+) and the same local oscillator signal that is supplied to the chip. The output signal was then acquired by a Bruker console and processed using the conventional TopSpin interface. 

**Figure 6:**
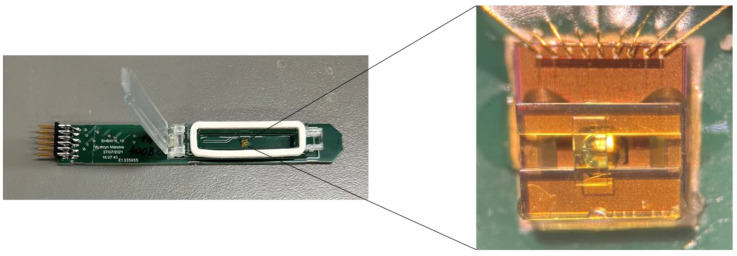
A photograph of the microchip PCB that connects to the main board, with an expansion showing the microstructure designed to hold a *D. magna* egg on top of the micro-coil.

The device was seated within a 3D printed probe phantom such that the coil could be reproducibly placed in the homogenous region of the magnet. The chip for coil 23B was designed with a microstructure optimized to seat a *D. magna* egg and was used for the ^1^H and ^19^F analysis of the egg. The coil is surrounded by a hydration chamber that is filled with water in order to prevent the egg from drying out during extended experiments. Both the microstructure and hydration chamber are pictured in Figure 6. Chip 30A was made without an egg holder to allow a wider variety of samples to be studied and was used to analyze standard solutions and the broccoli seed, which were held in thin-walled capillaries affixed to the coil using polytetrafluoroethylene tape.

In order to establish limits of detection for different heteronuclei, ten different nuclei were studied: ^1^H, ^7^Li, ^11^B, ^19^F, ^23^Na, ^31^P, ^27^Al, ^55^Mn, ^59^Co and ^205^Tl. This was carried out using the following standard solutions all dissolved in H_2_O: 0.15 M sodium acetate (Millipore Sigma) (^1^H), 1 M LiCl (Science Company) (^7^Li), 0.5 M H_3_BO_3_ (Fisher Scientific) (^11^B), neat trifluoroacetic acid (Millipore Sigma) (^19^F), 1 M NaH_2_PO_4_ (Fisher Scientific) (^23^Na, ^31^P), 0.65 M Al(NO_3_)_3_ (Fisher Scientific) (^27^Al), 0.45 M KMnO_4_ (Millipore Sigma) (^55^Mn), 0.25 M Co(NH_3_)_6_Cl_3_ (Millipore Sigma) (^59^Co) and 0.36 M TlNO_3_ (Millipore Sigma) (^205^Tl). The most important acquisition and processing parameters for the NMR standards can be found in Table 2. Data processing was performed using TopSpin 3.5.6 and MestreNova 12.4. Each spectrum was zero filled by a factor of 2 and multiplied by an exponential function corresponding to the line broadening found in Table 2. 

For ^13^C experiments, a single ^13^C labelled broccoli seed (*Brassica oleracea*, IsoLife) was placed in water for one week in order to induce sprouting and was then placed in a thin-walled capillary and analyzed on the micro-coil system. In order to improve the signal-to-noise ratio, the steady state free precession (SSFP) sequence [52,70] was used. This is a technique that has recently received renewed attention [52,71] and works by employing very short pulse-acquisition blocks, allowing for a much larger number of transients to be acquired. This can result in significant signal-to-noise improvements. The SSFP spectrum was acquired using 7,731,856 pulse acquire blocks with 1024 time domain points (corresponding to an acquisition time of ~17 ms) and a recycle delay of 11 ms. To examine the improvement in signal-to-noise ratio, a ^13^C spectrum was acquired using “standard” parameters for comparison. The standard spectrum was acquired using 10,240 scans, 32,768 time domain points and a recycle delay of 3 s, and compared to an SSFP spectrum acquired in the same amount of time. To allow a fair comparison, the standard spectrum was truncated to the same number of points as the SSFP spectrum, such that the noise towards the FID’s tail would not unfairly bias the signal-to-noise ratio. Following this, the SSFP spectra were zero filled by a factor of 4 and apodization was performed using a trapezoidal function. The ^1^H spectrum of the broccoli seed was acquired using 64 scans, 16,384 time domain points and a recycle delay of 1.5 s. 

For the ^19^F tracking experiments, a single *Daphnia magna* egg was exposed to a fluorinated contaminant: either hexafluorobenzene (Millipore Sigma) or trifluorotoluene (Millipore Sigma). A layer of water was placed over the liquid contaminant and the egg was placed in the water layer and left over night. The next morning, the egg was rinsed 3 times and placed into fresh water inside the micro-coil sample holder. The sample holder consists of a 3D printed microstructure optimized to seat an egg perfectly over the coil and is surrounded by a chamber filled with water to ensure that the egg does not dry out during longer experiments (see Figure 6). A ^19^F experiment was run every 6.3 min until the signal was no longer detectable. The ^19^F contaminant tracking experiments were acquired using 256 scans, 16,384 time domain points and a recycle delay of 1 s. Each spectrum was zero filled by a factor of 2 and multiplied by an exponential function corresponding to a line broadening of 25 Hz in the frequency domain. The ^1^H spectrum of the *D. magna* egg was acquired on a fresh egg using 122,840 scans, 16,384 time domain points and a recycle delay of 1 s. The spectrum was zero filled by a factor of 2 and multiplied by an exponential function corresponding to a line broadening of 5 Hz in the frequency domain.

## 4. Conclusions and Outlook

Here, the potential of a broadband CMOS micro-coil system for environmental research was explored. Eleven different nuclei were studied using the system and the limits of detection for an extended experiment were found to be in the picomole range. Environmental applications of these nuclei range from the study of lithium-ion battery leachates to persistent fluorinated pollutants and the examination of biochemical energy molecules. ^13^C NMR was then used to examine the metabolic profile of a ^13^C enriched broccoli seed, where the additional spectral dispersion compared to ^1^H NMR was utilized to help overcome the overlap caused by magnetic susceptibility distortions. It was also found that the use of the ^13^C SSFP sequence was very effective, giving a 6-fold signal-to-noise improvement with minimal resolution loss. Finally, as a proof of concept, a single *D. magna* egg was exposed to a fluorinated contaminant and the ^19^F NMR signal of the compound present inside the egg was tracked over time, showing the potential for studying pollutant-egg interactions for a variety of fluorinated compounds. 

Initially, the question posed was whether CMOS micro-coils can be useful for environmental research, and based on the results presented here, the answer is indeed “yes”. Further, there is considerable future potential to be explored using this technology. Since no tuning and matching is required, it is possible to run nearly any combination of experiments using a single channel. For example, during a standard ^13^C experiment where the recycle delay may be long, a spectrum of one or several heteronuclei such as ^19^F or ^31^P could be acquired, improving the information content of the experiment with minimal downside. Further, if the micro-coil is placed within a larger resonator and an external coil is used for pulsing, the B_1_ field homogeneity can be substantially improved, opening the door for more advanced experiments including gradients and multiple pulses. Though this would require modification of a standard NMR probe, there is significant potential for this approach. Finally, due to the manufacturing process of the micro-coils, it is relatively easy to scale up to an array of multiple coils and channels. This would allow the study of multiple eggs at one time, not only improving sample throughput, which remains a challenge, but also allowing for the study of exposed and control samples at the same time. This can help reduce natural variability and improve confidence in the results of toxicity/exposure studies. Overall, CMOS micro-coils have considerable potential for the study of mass limited samples within an environmental context. 

## Figures and Tables

**Table 1:** Detection limits and lineshape for the nuclei studied.

Nucleus	Limit of Detection (pmol)	Lineshape (Hz)
^1^H	15	4
^7^Li	72	3
^11^B	313	100
^19^F	19	10
^23^Na	253	19
^27^Al	296	21
^31^P	454	25
^55^Mn	272	50
^59^Co	251	189
^205^Tl	172	31

**Table 2:** Important acquisition and processing parameters for each nucleus studied in the standards.

Nucleus	^1^H	^7^Li	^11^B	^19^F	^23^Na	^27^Al	^31^P	^55^Mn	^59^Co	^205^Tl
TD	32,768	32,768	16,384	32,768	16,384	16,384	32,768	16,384	8192	16,384
NS	32	256	3072	1	256	98,304	256	15,360	72,704	32,768
D1 (s)	3	5	1.5	3	1	0.03	3	0.03	0.11	0.03
Line Broadening (Hz)	1	1	25	5	5	15	10	15	25	10

## Data Availability

The data presented in this study are available on request from the corresponding author.

## Supplementary Materials

The supporting information can be downloaded at: https://www.mdpi.com/article/10.3390/molecules28135080/s1. Figure S1: This consists of Heteronuclear NMR spectra on a range of chemical standards acquired at 11.7 T (500 MHz ^1^H) using the CMOS micro-coil device.

## References

[1] Maggio R.M., Calvo N.L., Vignaduzzo S.E., Kaufman T.S. (2014). Pharmaceutical Impurities and Degradation Products: Uses and Applications of NMR Techniques. J. Pharm. Biomed. Anal..

[2] Spraul M., Schütz B., Humpfer E., Mörtter M., Schäfer H., Koswig S., Rinke P. (2009). Mixture Analysis by NMR as Applied to Fruit Juice Quality Control. Magn. Reson. Chem..

[3] Anaraki M.T., Lysak D.H., Soong R., Simpson M.J., Spraul M., Bermel W., Heumann H., Gundy M., Boenisch H., Simpson A.J. (2020). NMR Assignment of the: In Vivo Daphnia Magna Metabolome. Analyst.

[4] Kovacs H., Moskau D., Spraul M. (2005). Cryogenically Cooled Probes—A Leap in NMR Technology. Prog. Nucl. Magn. Reson. Spectrosc..

[5] Styles P., Soffe N.F., Scott C.A., Cragg D.A., Row F., White D.J., White P.C.J. (2011). A High-Resolution NMR Probe in Which the Coil and Preamplifier Are Cooled with Liquid Helium. J. Magn. Reson..

[6] Wikus P., Frantz W., Kümmerle R., Vonlanthen P. (2022). Commercial Gigahertz-Class NMR Magnets. Supercond. Sci. Technol..

[7] Eills J., Budker D., Cavagnero S., Chekmenev E.Y., Elliott S.J., Jannin S., Lesage A., Matysik J., Meersmann T., Prisner T. (2023). Spin Hyperpolarization in Modern Magnetic Resonance. Chem. Rev..

[8] Webb A.G. (2008). Microcoil Nuclear Magnetic Resonance Spectroscopy. NMR Spectroscopy in Pharmaceutical Analysis.

[9] Fugariu I., Soong R., Lane D., Fey M., Maas W., Vincent F., Beck A., Schmidig D., Treanor B., Simpson A.J. (2017). Towards Single Egg Toxicity Screening Using Microcoil NMR. Analyst.

[10] Olson D.L., Lacey M.E., Sweedler J.V. (1998). Microcoils Significantly Boost NMR Mass Sensitivity and Provide New Detection Opportunities.: The Nanoliter Niche. Anal. Chem..

[11] van Bentum P.J.M., Janssen J.W.G., Kentgens A.P.M., Bart J., Gardeniers J.G.E. (2007). Stripline Probes for Nuclear Magnetic Resonance. J. Magn. Reson..

[12] Grisi M., Vincent F., Volpe B., Guidetti R., Harris N., Beck A., Boero G. (2017). NMR Spectroscopy of Single Sub-NL Ova with Inductive Ultra-Compact Single-Chip Probes. Sci. Rep..

[13] Bastawrous M., Gruschke O., Soong R., Jenne A., Gross D., Busse F., Nashman B., Lacerda A., Simpson A.J. (2022). Comparing the Potential of Helmholtz and Planar NMR Microcoils for Analysis of Intact Biological Samples. Anal. Chem..

[14] Moxley-Paquette V., Lane D., Soong R., Ning P., Bastawrous M., Wu B., Pedram M.Z., Haque Talukder M.A., Ghafar-Zadeh E., Zverev D. (2020). 5-Axis CNC Micromilling for Rapid, Cheap, and Background-Free NMR Microcoils. Anal. Chem..

[15] Persoone G., Baudo R., Cotman M., Blaise C., Thompson K.C., Moreira-Santos M., Vollat B., Törökne A., Han T. (2009). Review on the Acute Daphnia Magna Toxicity Test? Evaluation of the Sensitivity and the Precision of Assays Performed with Organisms from Laboratory Cultures or Hatched from Dormant Eggs. Knowl. Manag. Aquat. Ecosyst..

[16] De Coen W.M., Janssen C.R. (1998). The Use of Biomarkers in Daphnia Magna Toxicity Testing. Hydrobiologia.

[17] Nasser F., Lynch I. (2019). Updating Traditional Regulatory Tests for Use with Novel Materials: Nanomaterial Toxicity Testing with Daphnia Magna. Saf. Sci..

[18] Edison A.S., Hall R.D., Junot C., Karp P.D., Kurland I.J., Mistrik R., Reed L.K., Saito K., Salek R.M., Steinbeck C. (2016). The Time Is Right to Focus on Model Organism Metabolomes. Metabolites.

[19] Doma S. (1979). Ephippia of Daphnia Magna Straus—A Technique for Their Mass Production and Quick Revival. Hydrobiologia.

[20] De Meester L., De Jager H. (1993). Hatching of Daphnia Sexual Eggs. II. The Effect of Age and a Second Stimulus. Freshw. Biol..

[21] Poynton H.C., Varshavsky J.R., Chang B., Cavigiolio G., Chan S., Holman P.S., Loguinov A.V., Bauer D.J., Komachi K., Theil E.C. (2007). Daphnia Magna Ecotoxicogenomics Provides Mechanistic Insights into Metal Toxicity. Environ. Sci. Technol..

[22] Cuenca Cambronero M., Marshall H., De Meester L., Davidson T.A., Beckerman A.P., Orsini L. (2018). Predictability of the Impact of Multiple Stressors on the Keystone Species Daphnia. Sci. Rep..

[23] Lysak D.H., Kock F.V.C., Mamone S., Soong R., Glöggler S., Simpson A.J. (2023). In Vivo Singlet State Filtered Nuclear Magnetic Resonance: Towards Monitoring Toxic Responses inside Living Organisms. Chem. Sci..

[24] Krewski D., Acosta D., Andersen M., Anderson H., Bailar J.C., Boekelheide K., Brent R., Charnley G., Cheung V.G., Green S. (2010). Staff of Committee on Toxicity Test. Toxicity Testing in the 21st Century: A Vision and a Strategy. J. Toxicol. Environ. Health Part B.

[25] Barata C., Alañon P., Gutierrez-Alonso S., Riva M.C., Fernández C., Tarazona J.V. (2008). A Daphnia Magna Feeding Bioassay as a Cost Effective and Ecological Relevant Sublethal Toxicity Test for Environmental Risk Assessment of Toxic Effluents. Sci. Total Environ..

[26] Grisi M. (2017). Broadband Single-Chip Transceivers for Compact NMR Probes.

[27] Grisi M., Conley G.M., Rodriguez K.J., Riva E., Egli L., Moritz W., Lichtenberg J., Brugger J., Boero G. (2020). NMR Microsystem for Label-Free Characterization of 3D Nanoliter Microtissues. Sci. Rep..

[28] Grisi M., Gualco G., Boero G. (2015). A Broadband Single-Chip Transceiver for Multi-Nuclear NMR Probes. Rev. Sci. Instrum..

[29] Anders J., Dreyer F., Krüger D., Schwartz I., Plenio M.B., Jelezko F. (2021). Progress in Miniaturization and Low-Field Nuclear Magnetic Resonance. J. Magn. Reson..

[30] Grisi M., Conley G.M. (2020). Cmos-Based Sensors as New Standard for Micro-Nmr: Magnetic Resonance at the Embryo Scale. eMagRes.

[31] Anders J., Korvink J.G. (2018). Micro and Nano Scale NMR.

[32] Anders J., Chiaramonte G., SanGiorgio P., Boero G. (2009). A Single-Chip Array of NMR Receivers. J. Magn. Reson..

[33] Yang Q., Zhao J., Dreyer F., Krüger D., Anders J. (2022). A Portable NMR Platform with Arbitrary Phase Control and Temperature Compensation. Magn. Reson..

[34] Sivelli G., Conley G.M., Herrera C., Marable K., Rodriguez K.J., Bollwein H., Sudano M.J., Brugger J., Simpson A.J., Boero G. (2022). NMR Spectroscopy of a Single Mammalian Early Stage Embryo. J. Magn. Reson..

[35] Kim T., Song W., Son D.-Y., Ono L.K., Qi Y. (2019). Lithium-Ion Batteries: Outlook on Present, Future, and Hybridized Technologies. J. Mater. Chem. A.

[36] Mrozik W., Ali Rajaeifar M., Heidrich O., Christensen P. (2021). Environmental Impacts, Pollution Sources and Pathways of Spent Lithium-Ion Batteries. Energy Environ. Sci..

[37] Stamp A., Lang D.J., Wäger P.A. (2012). Environmental Impacts of a Transition toward E-Mobility: The Present and Future Role of Lithium Carbonate Production. J. Clean. Prod..

[38] Brown T.R., Ugurbil K., Shulman R.G. (1977). 31P Nuclear Magnetic Resonance Measurements of ATPase Kinetics in Aerobic Escherichia Coli Cells. Proc. Natl. Acad. Sci. USA.

[39] de Graaf R.A., van Kranenburg A., Nicolay K. (2000). In Vivo 31P-NMR Diffusion Spectroscopy of ATP and Phosphocreatine in Rat Skeletal Muscle. Biophys. J..

[40] Sundareshwar P.V., Morris J.T., Pellechia P.J., Cohen H.J., Porter D.E., Jones B.C. (2001). Occurrence and Ecological Implications of Pyrophosphate in Estuaries. Limnol. Oceanogr..

[41] Clark L.L., Ingall E.D., Benner R. (1999). Marine Organic Phosphorus Cycling; Novel Insights from Nuclear Magnetic Resonance. Am. J. Sci..

[42] Turner B.L., Newman S. (2005). Phosphorus Cycling in Wetland Soils. J. Environ. Qual..

[43] Galván-Arzate S., Santamaría A. (1998). Thallium Toxicity. Toxicol. Lett..

[44] Weaver C.D., Harden D., Dworetzky S.I., Robertson B., Knox R.J. (2004). A Thallium-Sensitive, Fluorescence-Based Assay for Detecting and Characterizing Potassium Channel Modulators in Mammalian Cells. SLAS Discov..

[45] Douglas K.T., Bunni M.A., Baindur S.R. (1990). Thallium in Biochemistry. Int. J. Biochem..

[46] Harris R.K., Becker E.D., Cabral de Menezes S.M., Goodfellow R., Granger P. (2001). NMR Nomenclature. Nuclear Spin Properties and Conventions for Chemical Shifts. Pure Appl. Chem..

[47] Ashbrook S.E. (2009). Recent Advances in Solid-State NMR Spectroscopy of Quadrupolar Nuclei. Phys. Chem. Chem. Phys..

[48] Anaraki M.T., Lysak D.H., Downey K., Kock F.V.C., You X., Majumdar R.D., Barison A., Lião L.M., Ferreira A.G., Decker V. (2021). NMR Spectroscopy of Wastewater: A Review, Case Study, and Future Potential. Prog. Nucl. Magn. Reson. Spectrosc..

[49] Subramanian R., Lam M.M., Webb A.G. (1998). RF Microcoil Design for Practical NMR of Mass-Limited Samples. J. Magn. Reson..

[50] Bieri O., Scheffler K. (2013). Fundamentals of Balanced Steady State Free Precession MRI. J. Magn. Reson. Imaging.

[51] Murphy D.J. (2016). Plant Storage Lipids. Encyclopedia of Life Sciences.

[52] Moraes T.B., Kock F.V.C., Salome K.S., Barison A., Simpson A., Colnago L.A. (2023). Steady-State Free Precession Sequences for High and Low Field NMR Spectroscopy in Solution: Challenges and Opportunities. J. Magn. Reson. Open.

[53] Mobarhan Y.L., Struppe J., Fortier-McGill B., Simpson A.J. (2017). Effective Combined Water and Sideband Suppression for Low-Speed Tissue and in Vivo MAS NMR. Anal. Bioanal. Chem..

[54] Taipale S.J., Kainz M.J., Brett M.T. (2011). Diet-Switching Experiments Show Rapid Accumulation and Preferential Retention of Highly Unsaturated Fatty Acids in Daphnia. Oikos.

[55] Martin-Creuzburg D., von Elert E. (2009). Good Food versus Bad Food: The Role of Sterols and Polyunsaturated Fatty Acids in Determining Growth and Reproduction of Daphnia Magna. Aquat. Ecol..

[56] Masclaux H., Bec A., Kainz M.J., Perrière F., Desvilettes C., Bourdier G. (2012). Accumulation of Polyunsaturated Fatty Acids by Cladocerans: Effects of Taxonomy, Temperature and Food. Freshw. Biol..

[57] Becker C., Boersma M. (2005). Differential Effects of Phosphorus and Fatty Acids on Daphnia Magna Growth and Reproduction. Limnol. Oceanogr..

[58] Hakumäki J.M., Poptani H., Sandmair A.-M., Ylä-Herttuala S., Kauppinen R.A. (1999). 1H MRS Detects Polyunsaturated Fatty Acid Accumulation during Gene Therapy of Glioma: Implications for the in Vivo Detection of Apoptosis. Nat. Med..

[59] Glüge J., Scheringer M., Cousins I.T., DeWitt J.C., Goldenman G., Herzke D., Lohmann R., Ng C.A., Trier X., Wang Z. (2020). An Overview of the Uses of Per- and Polyfluoroalkyl Substances (PFAS). Environ. Sci. Process. Impacts.

[60] Brunn H., Arnold G., Körner W., Rippen G., Steinhäuser K.G., Valentin I. (2023). PFAS: Forever Chemicals—Persistent, Bioaccumulative and Mobile. Reviewing the Status and the Need for Their Phase out and Remediation of Contaminated Sites. Environ. Sci. Eur..

[61] Podder A., Sadmani A.H.M.A., Reinhart D., Chang N.-B., Goel R. (2021). Per and Poly-Fluoroalkyl Substances (PFAS) as a Contaminant of Emerging Concern in Surface Water: A Transboundary Review of Their Occurrences and Toxicity Effects. J. Hazard. Mater..

[62] Young C.J., Furdui V.I., Franklin J., Koerner R.M., Muir D.C.G., Mabury S.A. (2007). Perfluorinated Acids in Arctic Snow:  New Evidence for Atmospheric Formation. Environ. Sci. Technol..

[63] Miner K.R., Clifford H., Taruscio T., Potocki M., Solomon G., Ritari M., Napper I.E., Gajurel A.P., Mayewski P.A. (2021). Deposition of PFAS ‘Forever Chemicals’ on Mt. Everest. Sci. Total Environ..

[64] Jian J.-M., Chen D., Han F.-J., Guo Y., Zeng L., Lu X., Wang F. (2018). A Short Review on Human Exposure to and Tissue Distribution of Per- and Polyfluoroalkyl Substances (PFASs). Sci. Total Environ..

[65] Murphy C.D. (2016). Microbial Degradation of Fluorinated Drugs: Biochemical Pathways, Impacts on the Environment and Potential Applications. Appl. Microbiol. Biotechnol..

[66] Shirzadi A., Simpson M.J., Xu Y., Simpson A.J. (2008). Application of Saturation Transfer Double Difference NMR to Elucidate the Mechanistic Interactions of Pesticides with Humic Acid. Environ. Sci. Technol..

[67] Buchholz C.R., Pomerantz W.C.K. (2021). 19F NMR Viewed through Two Different Lenses: Ligand-Observed and Protein-Observed 19F NMR Applications for Fragment-Based Drug Discovery. RSC Chem. Biol..

[68] Jan Hendriks A. (1995). Modelling Non-Equilibrium Concentrations of Microcontaminants in Organisms: Comparative Kinetics as a Function of Species Size and Octanol-Water Partitioning. Chemosphere.

[69] Shirzadi A., Simpson M.J., Kumar R., Baer A.J., Xu Y., Simpson A.J. (2008). Molecular Interactions of Pesticides at the Soil−Water Interface. Environ. Sci. Technol..

[70] Carr H.Y. (1958). Steady-State Free Precession in Nuclear Magnetic Resonance. Phys. Rev..

[71] Wolf T., Jaroszewicz M.J., Frydman L. (2021). Steady-State Free Precession and Solid-State NMR: How, When, and Why. J. Phys. Chem. C.

