# Supplementary material for: Exploring the Potential of Broadband Complementary Metal Oxide Semiconductor Micro-Coil Nuclear Magnetic Resonance for Environmental Research
Source: Molecules. 2023 Jun 29;28(13):5080. doi: 10.3390/molecules28135080 (PMC10343494; doi:10.3390/molecules28135080)
Supplement: Supplementary file 1 [file molecules-28-05080-s001.zip › molecules-2455572-supplementary.pdf]

Supporting information

Exploring the Potential of Broadband Complementary Metal Oxide Semiconductor Micro-Coil Nuclear Magnetic Resonance for Environmental Research

Daniel H. Lysak[1], Marco Grisi[2], Kathryn Marable[2], Gaurasundar M. Conley[2], Carl A. Michal[3], Vincent Moxley-Paquette[1], William W. Wolff[1], Katelyn Downey[1], Flavio V. C. Kock[1], Peter M. Costa[1], Kiera Ronda[1], Tiago B. Moraes[4], Katrina Steiner[1], Luiz A. Colnago[5] and Andre J. Simpson[1]

[1]Environmental NMR Centre, University of Toronto, Toronto, ON, M1C 1A4, Canada
[2]Annaida Technologies, Innovation Park, Lausanne, 1015, Switzerland
[3]Department of Physics and Astronomy, University of British Columbia, Vancouver, BC, V6T 1Z1,Canada
[4]Departamento Engenharia de Biossistemas, Universidade de São Paulo/ESALQ, Av. Páduas Dias, 11, Piracicaba, SP, 13418-900, Brazil
[5]Embrapa Instrumentação, Rua XV de Novembro 1452, São Carlos, SP, 13560-970, Brazil
*Correspondence: andre.simpson@utoronto.ca

Table of Contents

S1. Heteronuclear NMR Spectra S2

S1. Heteronuclear NMR Spectra

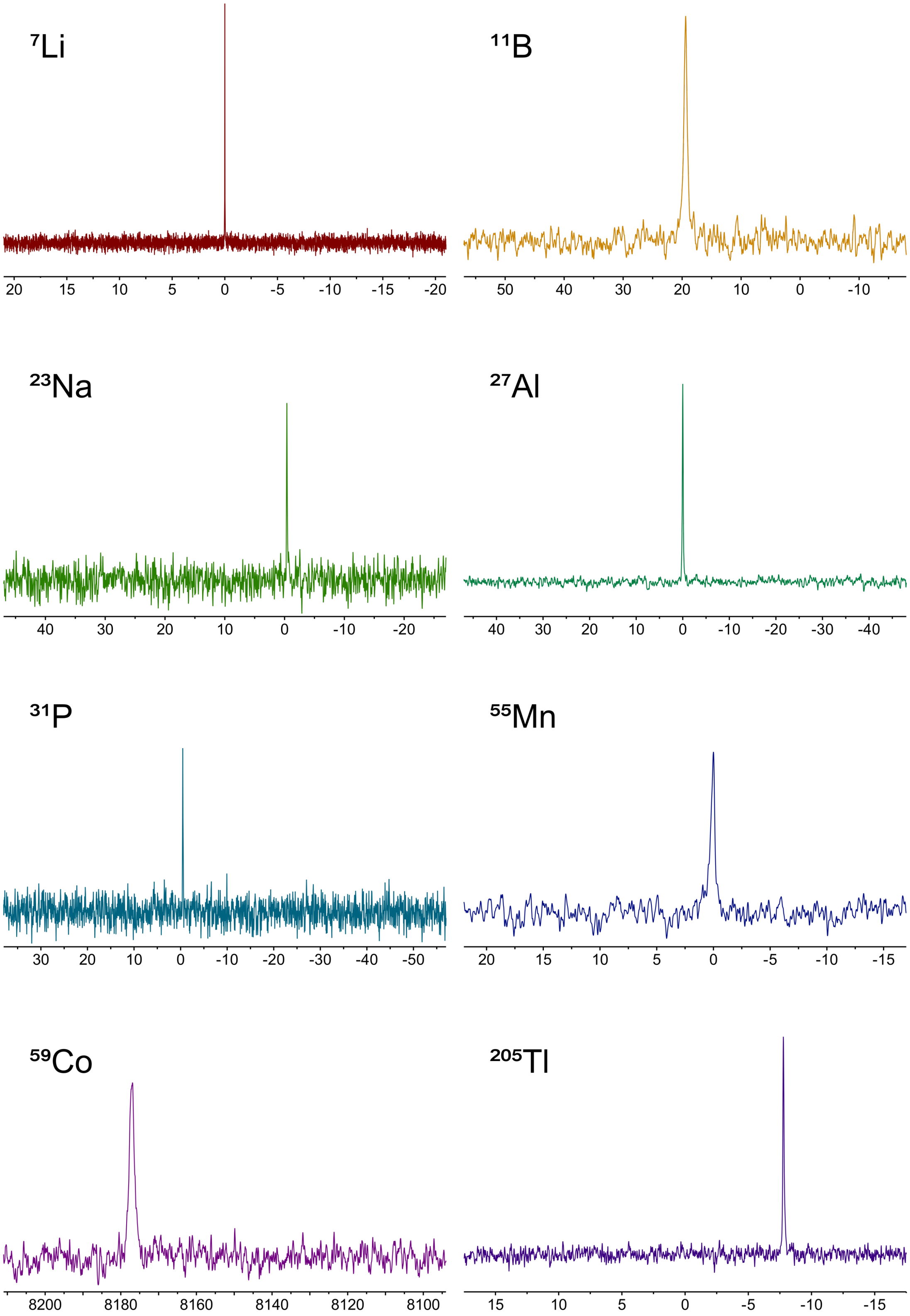

Figure S1. Heteronuclear NMR spectra on a range of chemical standards acquired at 11.7 T (500 MHz ^{1}H) using the CMOS micro-coil device. See the Materials and Methods section for acquisition and processing parameters.
